# Baseline and Impact of First-Year Intervention on *Schistosoma haematobium* Infection in Seasonal Transmission Foci in the Northern and Central Parts of Côte d’Ivoire

**DOI:** 10.3390/tropicalmed6010007

**Published:** 2021-01-08

**Authors:** Nana R. Diakité, Mamadou Ouattara, Fidèle K. Bassa, Jean T. Coulibaly, Yves-Nathan T. Tian-Bi, Aboulaye Meïté, Jan Hattendorf, Jürg Utzinger, Eliézer K. N’Goran

**Affiliations:** 1 Unité de Formation et de Recherche Biosciences, Université Félix Houphouët-Boigny, Abidjan 22 BP 770, Ivory Coast; mamadou_ouatt@yahoo.fr (M.O.); fidelebassa@gmail.com (F.K.B.); couljeanvae@yahoo.fr (J.T.C.); nathantianbi@gmail.com (Y.-N.T.T.-B.); eliezerngoran@yahoo.fr (E.K.N.); 2 Centre Suisse de Recherches Scientifiques en Côte d’Ivoire, Abidjan 01 BP 1303, Ivory Coast; 3 Department of Epidemiology and Public Health, Swiss Tropical and Public Health Institute, P.O. Box, CH-4002 Basel, Switzerland; jan.hattendorf@swisstph.ch (J.H.); juerg.utzinger@swisstph.ch (J.U.); 4 Faculty of Science, University of Basel, P.O. Box, CH-4003 Basel, Switzerland; 5 Programme National de Lutte contre les Maladies Tropicales Négligées à Chimiothérapie Préventive, Abidjan 06 BP 6394, Ivory Coast; aboulaye_meite77@yahoo.fr

**Keywords:** *Schistosoma haematobium*, control strategies, prevalence, intensity, seasonal transmission, Côte d’Ivoire

## Abstract

In order to assess the impact of different control strategies against *Schistosoma haematobium* in seasonal transmission foci in Côte d’Ivoire, a three-year cluster randomized trial was implemented. The decrease in *S. haematobium* prevalence among children aged 9−12 years was the primary outcome. In the first step, an eligibility survey was conducted, subjecting 50 children aged 13−14 years to a single urine filtration. Sixty-four villages with a prevalence of *S. haematobium* of ≥4% were selected and randomly assigned to four intervention arms consisting of annual mass drug administration (MDA) before (arm 1) and after (arm 2) the peak transmission, biannual treatment with praziquantel before and after the peak transmission season (arm 3), and annual MDA before the peak transmission season, coupled with focal chemical snail control using molluscicides (arm 4). At baseline, we observed a prevalence of 24.8%, 10.1%, 13.9%, and 15.9% in study arms 1, 2, 3, and 4, respectively. One year after the first intervention, the prevalence decreased in all study arms by about two-thirds or more. The prevalence in arm 2 was lower than in arm 1 (3.5% vs. 8.1%), but the difference was not statistically significant (odds ratio (OR) = 0.42, 95% confidence interval (CI) = 0.10−1.80). After adjusting for baseline prevalence, arms 1 and 2 performed roughly similarly (adjusted odds ratio (aOR) = 1.03, 95% CI = 0.34−3.07). The prevalence in arms 3 and 4 (1.9% and 2.2%) were significantly lower compared to arm 1 in the unadjusted and the adjusted models (arm 3 vs. arm 1, OR = 0.22, 95% CI = 0.05−0.95, aOR = 0.19, 95% CI = 0.08−0.48; arm 4 vs. arm 1, OR = 0.26, 95% CI = 0.08−0.85, aOR = 0.23, 95% CI = 0.06−0.87). The initial intervention showed a significant impact on the prevalence of *S. haematobium*. It will be interesting to monitor the comparative impact of the different intervention arms and to determine whether the interruption of seasonal transmission of *S. haematobium* can be achieved in this epidemiological setting within three years.

## 1. Introduction

Schistosomiasis is a neglected tropical disease that remains a public health problem in many parts of the tropics and subtropics [1,2]. Approximately 800 million people are at risk of schistosomiasis, with more than 250 million people infected [3]. In 2017, the estimated global burden of schistosomiasis was 1.9 million disability-adjusted life years (DALYs) [4]. The World Health Organization (WHO) has set ambitious goals of controlling morbidity to be reached by 2020, along with elimination as a public health problem in certain regions by 2025. Noting considerable progress made in schistosomiasis control in some regions, at the 65th World Health Assembly (WHA) in 2012, all endemic countries were called upon to intensify control interventions and strengthen surveillance [5]. In adopting resolution WHA 65.21, member states also requested the WHO to mobilize resources required to support integrated and multi-sectoral control programmes [6,7]. In line with WHO guidelines to control morbidity and eventually interrupt transmission, the Schistosomiasis Consortium for Operational Research and Evaluation (SCORE) launched several large-scale studies to gain and sustain schistosomiasis control and to eliminate disease transmission [8,9,10].

We carried out this study to assess different approaches aimed at achieving *Schistosoma haematobium* elimination goals in marked seasonal transmission foci in northern and central parts of Côte d’Ivoire. The overarching aim is to assess the impact of different interventions, namely (i) annual mass drug administration (MDA) before the peak transmission season; (ii) annual MDA after the peak transmission season; (iii) biannual treatment before and after the peak of schistosomiasis transmission season; and (iv) annual MDA before the peak of schistosomiasis transmission season, coupled with focal snail control using niclosamide [11]. Here, we report the baseline data and changes in the prevalence and intensity of *S. haematobium* infection one year after the initial intervention.

## 2. Materials and Methods

### 2.1. Ethics Statement

The study obtained ethical clearance from the national ethics committee in Côte d’Ivoire (reference no. 113/MSLS/CNER-dkn) and authorization for the use of niclosamide by the Comité Pesticide National, Direction Générale des Productions et de la Sécurité Alimentaire, Ministère de l’Agriculture (reference no. 0163/MINAGRI/DGPSA/DPVCQ). Additionally, the study was cleared by the ethics committee of Nord-West and Central Switzerland (EKNZ; reference no. UBE-15/34). Approval was also granted by local administrative, health, and village authorities of the six participating regions. Village committees were informed of the objectives, procedures, and potential risks and benefits of the study. Written informed consent was obtained from adult participants and from parents/guardians of minors (individuals aged < 18 years).

At the end of the baseline parasitological survey, individuals aged 5 years and above of the 64 randomized villages were invited to receive MDA using praziquantel (40 mg/kg), provided free of charge by the Programme National de Lutte contre les Maladies Tropicales Négligées à Chimiothérapie Préventive (PNLMTN-CP).

### 2.2. Study Area

**Figure 1:**
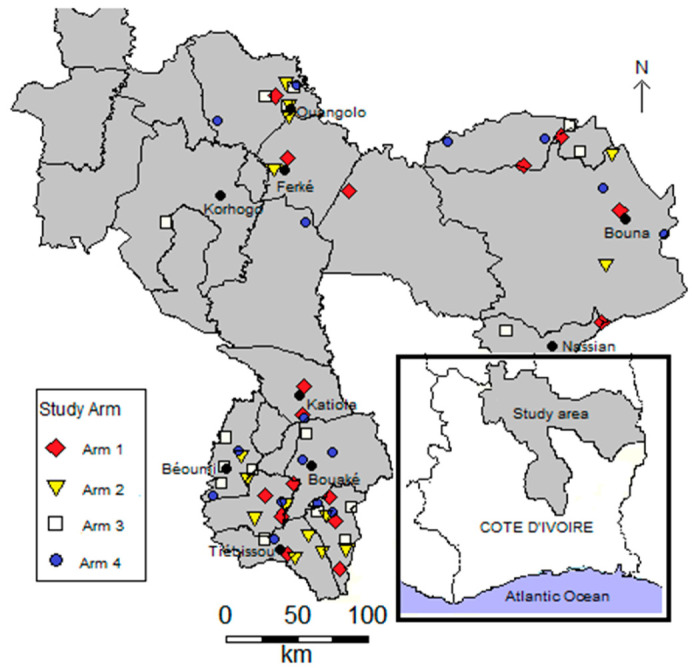
Map of the study area showing villages by arm in the northern and central parts of Côte d’Ivoire (n = 64).

Côte d’Ivoire is a country located in West Africa, composed of 31 administrative regions. The 64 participating villages are located in six regions, four situated in the northern (Tchologo, Poro, Hambol, and Bounkani) and two in the central part of Côte d’Ivoire (Gbêkê and Belier). Figure 1 shows a map of the study area, depicting the distribution of villages by study arms.

The climate is characterized by two well-defined seasons: the dry season, which last from November to March, and the rainy season from April to October. The mean annual precipitation is about 1000 mm in the northern part and ranges between 1200 and 1500 mm in the central part. In the 1970s and 1980s, several hundred small multipurpose dams were constructed to enhance agricultural production and livestock rearing. The scarcity of rains and concentration of water bodies during the dry season increases human and animal water contact, and hence, the transmission of schistosomiasis. A detailed description of the study area, including main activities and data on seasonal *S. haematobium* transmission, is available in the study protocol [11].

### 2.3. Study Design and Participants

The study was designed as a cluster-randomized trial. As with other SCORE-funded studies in Côte d’Ivoire and elsewhere in sub-Saharan Africa, the eligibility criteria were (i) the presence of a primary school with at least 100 enrolled children aged 9−12 years [8,9,11,12]; and (ii) prevalence of *S. haematobium* among 50 children aged 13−14 years between 4% and 10%, based on a single urine examination. Of note, school-aged children are at highest risk of schistosomiasis, and are the primary target group by large-scale control activities [1,2]. Additionally, 50 first-grade children aged 5−8 years and 50 adults aged 20−55 years were enrolled to determine the effect of different interventions on the force of transmission in the community. Data on first-grade children and adults were collected at the baseline and end-of-study cross-sectional surveys [11].

After surveying 122 villages without identifying enough eligible localities, the second inclusion criterion was relaxed, and villages with a prevalence of *S. haematobium* among children aged 13−14 years ≥ 4% were allowed to participate. A vast study area was prospected, and villages not only in the northern, as initially planned, but also in the central part of Côte d’Ivoire were included.

Taken together, 64 villages fulfilled the two inclusion criteria, and hence, they were randomly assigned to one of the four interventions arms: (i) arm 1: annual MDA with praziquantel before the peak transmission season; (ii) arm 2: annual MDA after the peak transmission season; (iii) arm 3: biannual MDA before and after the peak transmission season; and (iv) arm 4: annual MDA before the peak transmission season, coupled with chemical snail control using niclosamide (three applications per year, before, during, and shortly after peak transmission). Details on sample size have been published elsewhere [11].

### 2.4. Interventions

Praziquantel (40 mg/kg) was administered to the whole eligible population by the PNLMTN-CP. According to the study protocol, MDA used both approaches: school-based treatment (SBT) and community-wide treatment (CWT) [12,13]. A few days after the baseline survey, MDA was implemented from 14−26 December 2015 in arms 1, 3, and 4. From 7 to 12 April 2016, the other MDA was carried out in arm 2 and for the second time in arm 3. At each MDA time point, experienced medical staff observed for specific exclusion criteria (i.e., hypersensitivity to praziquantel, severely ill people, and pregnancy). Trained teachers treated pupils at school, while community health workers (CHWs) treated adults and non-enrolled children at home.

### 2.5. Parasitological Procedures

#### 2.5.1. Eligibility Survey

The cross-sectional eligibility survey was carried out over five months from May to September 2015. Overall, 208 villages were surveyed and 64 fulfilled the two inclusion criteria.

#### 2.5.2. Baseline and One-Year Follow-up Survey

The baseline survey was conducted from 9 to 28 November 2015. In each village, 100 children aged 9−12 years and 50 first-year students aged 5−8 years were randomly selected from the lists at school. In addition, 50 adults (aged 20−55 years) were selected from the community. For selection of adults, a central location within each village was identified, and from the centre, four different directions were determined. With the support of CHWs, houses in each direction were visited, and adults aged 20−55 years were invited to participate. This procedure was repeated until a total of 50 adults were recruited.

After obtaining written informed consent from adults and parents/guardians of minors (<18 years), a urine sample was collected from each participant between 09:00 and 14:00 hours in a plastic container labelled with unique identification. Urine samples were transferred to a nearby laboratory and examined for *S. haematobium* the same day. In brief, each urine sample was assessed for the presence of hematuria by visual detection of blood and urine reagent strip (Multistix®, Siemens Healthcare Diagnostics Inc., USA) testing for microhaematuria. The urinalysis was performed according to the manufacturer’s instructions.

The prevalence and intensity of *S. haematobium* infection were estimated based on the urine filtration method. In brief, 10 mL of urine was pressed through a polyamide (Nytrel) filter (mesh size: 20 μm) and stained with a drop of Lugol’s solution for subsequent microscopic examination. The intensity of *S. haematobium* infection was expressed as the number of eggs per 10 mL of urine [14].

### 2.6. Statistical Analysis

Data were entered in Excel (Microsoft Corporation; Redmond, USA), and cleaned and analyzed with SAS version 9.4 (SAS Institute Inc.; Cary, USA) and R version 5.3.1 (R Foundation for Statistical Computing; Vienna, Austria). A participant was considered infected with *S. haematobium* when at least one egg was observed in the urine sample subjected to a filtration method. The arithmetic mean of infection intensity was estimated at both the individual level (excluding negative children) and the village level (including negative children), expressed as the number of *S. haematobium* eggs per 10 mL of urine. The egg reduction rate (ERR) in *S. haematobium* infection was calculated as follows: ERR = (1−first follow-up infection intensity/baseline infection intensity) × 100. Infected participants with 1−49 eggs per 10 mL of urine were considered to have light infection, while ≥50 eggs per 10 mL of urine was considered as heavy infection, according to WHO guidelines [5,15]. Egg counts were truncated at 1000 eggs per 10 mL of urine.

Generalized estimating equation (GEE) models with binary and negative binomial distributed outcomes and independent correlation structure were applied to compare trial arms. All models used robust variance estimators to account for correlation within clusters. Comparison in prevalence and infection intensity between study arms was made at the one-year follow-up. Arm 1 was designated as the reference group. Adjusted odds ratios (aORs) were calculated using age, sex, and the cluster prevalence at baseline as additional covariates. The adjusted models were weighted to account for potential different numbers of samples collected in each cluster to guarantee that all clusters had the same weight in the analysis.

Coverage in the first MDA round with praziquantel in schoolchildren was calculated as the proportion of children who effectively participated in the SBT among all schoolchildren surveyed [13]. Coverage for adults was calculated as the proportion of adults who had received and swallowed praziquantel tablets among all surveyed adults.

Because we observed a considerable imbalance in prevalence among study arms at baseline, we conducted in addition to the statistical analysis plan an exploratory analysis using inverse probability weights to account for imbalances at baseline. The exploratory results are visualized in the supplementary appendix.

## 3. Results

### 3.1. Population Characteristics

**Figure 2:**
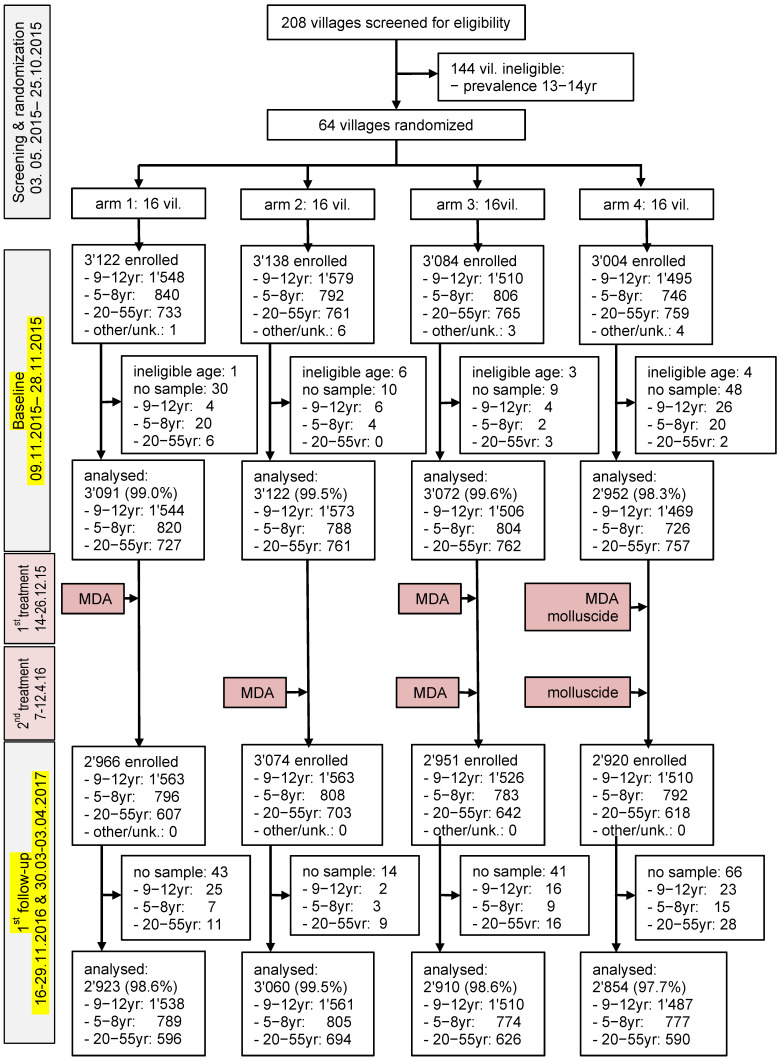
Study flow chart.

The baseline survey was conducted in the 64 villages from 9 to 28 November 2015. Figure 2 shows the study flow and baseline characteristics. Overall, 12,348 participants were enrolled. However, 111 were excluded from further analysis because they did not provide urine samples (n = 97) or were outside the requested age ranges (n = 14). Complete data records were available from 12,237 participants. There were 3138 first-grade children (1608 boys and 1530 girls), 6092 children aged 9−12 years (3423 boys and 2669 girls), and 3007 adults aged 20−55 years. The mean age of these three groups was 6.8 years, 10.3 years, and 37.3 years, respectively. The number of children aged 9−12 years in the different intervention arms was 1544 (25.3%) in arm 1, 1573 (25.8%) in arm 2, 1506 (24.7%) in arm 3, and 1469 (24.1%) in arm 4. The study population’s characteristics, stratified by study arm at baseline, are summarized in Table 1.

At the first follow-up survey, implemented one year after the initial intervention, 164 of the 11,911 participants did not provide urine samples. Hence, complete data records were available from 11,747 participants (males = 5911, females = 5836), and these were subjected to further statistical analyses (Figure 2).

### 3.2. S. haematobium Infection at Baseline

#### 3.2.1. Children Aged 9−12 Years

The overall *S. haematobium* prevalence at baseline was 16.2%. The prevalence in treatment arms 1, 2, 3, and 4 was 24.8%, 10.1%, 13.9%, and 18.8%, respectively. The prevalence of heavy intensity infection (i.e., ≥50 eggs per 10 mL of urine) ranged from 3.4% to 6.8% (Table 1). The prevalence of *S. haematobium* in boys was significantly higher than in girls (18.4% vs. 13.3%, χ^2^ = 29.6, *p* < 0.001).

The arithmetic mean (AM) egg counts per 10 mL of urine at baseline was 17.9, 5.7, 8.4, and 6.2 eggs per 10 mL of urine in arms 1, 2, 3, and 4, respectively. The prevalence showed considerable heterogeneity. While most of the villages presented low prevalence (<10%), six villages were classified as high risk communities (one each in arms 1 and 2, three in arm 3, and two in arm 4). With regard to the AM infection intensity, three villages pre-sented heavy infections (color orange to red; two in arm 1 and one in arm 2), with 73.9, 57.8, and 58.0 eggs per 10 mL of urine, respectively.

#### 3.2.2. First-Grade Children and Adults

**Figure 3:**
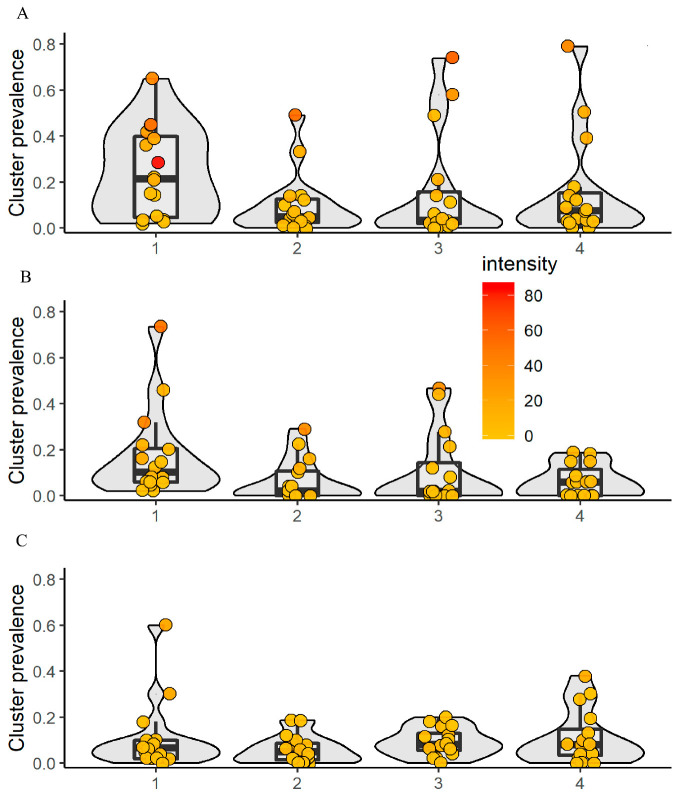
Village-level prevalence and intensity of *S. haematobium*, stratified by age group and intervention arm in 64 villages at baseline. The color code shows the mean infection intensity at the village level with low (yellow) and heavy (red). 1 = arm 1; 2 = arm 2; 3 = arm 3; 4 = arm 4. (**A**) Schoolchildren aged 9−12-years; (**B**) first-grade children aged 5−8 years; (**C**) adults aged 20−55 years.

Baseline data revealed that out of 3138 first-grade children, 10.6% were infected with *S. haematobium*. The prevalence in arms 1, 2, 3, and 4 was 17.9%, 6.3%, 10.8%, and 6.6%, respectively. For adults, out of the 3007 examined, 9.4% were infected with *S. haematobium*. The prevalence in the four intervention arms was 11.7%, 6.0%, 9.2%, and 11.0%, respectively. The proportion of heavily infected villages, stratified by study arm, ranged from 1.1% to 5.2% in first-grade children and from 1.0% to 1.9% in adults. There was no significant difference in the prevalence of first-grade children between boys and girls. The AM infection intensity of first-grade children was 9.6 eggs per 10 mL of urine in arm 1, 4.1 eggs per 10 mL of urine in arm 2, 4.5 eggs per 10 mL of urine in arm 3, and 2.0 eggs per 10 mL of urine in arm 4. For adults, the AM infection intensity in the four intervention arms was 4.0, 2.0, 1.1, and 2.1 eggs per 10 mL of urine, respectively. For first-grade children and adults, all four arms showed low prevalence when aggregated at the village level, except in arm 1 (two villages showing high prevalence; one in first-grade children and one in adults). In these age groups, no heavy infection intensities were recorded (Figure 3).

### 3.3. Differences Among Treatment Arms One Year After Intervention

#### 3.3.1. Children Aged 9−12 Years

At the one-year treatment follow-up, a prevalence of 3.5% was observed in arm 2, compared to 8.1% in arm 1 (OR = 0.42, 95% CI = 0.10−1.80). However, after adjusting for baseline prevalence, the difference was not statistically significant (aOR = 1.03, 95% CI = 0.34−3.07). The prevalence in arm 3 was 1.9%, and statistically significantly lower compared to arm 1 in the unadjusted analysis (OR = 0.22, 95% CI = 0.05−0.95) and adjusted analysis (aOR = 0.20, 95% CI = 0.08-0.48). A similar pattern was observed in arm 4 with a prevalence of 2.2% (OR = 0.26, 95% CI = 0.08−0.85; aOR = 0.23, 95% CI = 0.06−0.87). Of note, the prevalence decreased in all arms by about two-thirds or more.

#### 3.3.2. First-Grade Children

In first-grade children, we observed the same pattern as in their older counterparts. However, none of the observed between-arm differences was statistically significant. The prevalence in arm 2 was 2.6% compared to 5.2% in arm 1 (OR = 0.49, 95% CI = 0.08−3.01; aOR = 1.38, 95% CI = 0.17−11.1). The lowest prevalence was observed in arm 3, with 1.3% (OR = 0.24, 95% CI = 0.06−1.03; aOR = 0.39, 95% CI = 0.10−1.54). The decline in prevalence from baseline ranged from 59% in arm 2 to 88% in arms 3 and 4.

#### 3.3.3. Adults

At the one-year treatment follow-up, a prevalence of 1.9% was observed in adults in arm 2, which differed only slightly from the 3.0% in arm 1. In arm 3, the prevalence was 0.8%, which was statistically significantly different from arm 1 in the unadjusted and adjusted analyses (OR = 0.26, 95% CI = 0.08−0.81; aOR = 0.32, 95% CI = 0.11−0.96). The lowest prevalence was observed in arm 4, which differed significantly from arm 1 both in the unadjusted (OR = 0.22, 95% CI = 0.06−0.79) and adjusted analyses (aOR = 0.21, 95% CI = 0.07−0.64) (Table 2 and Table 3).

**Figure 4:**
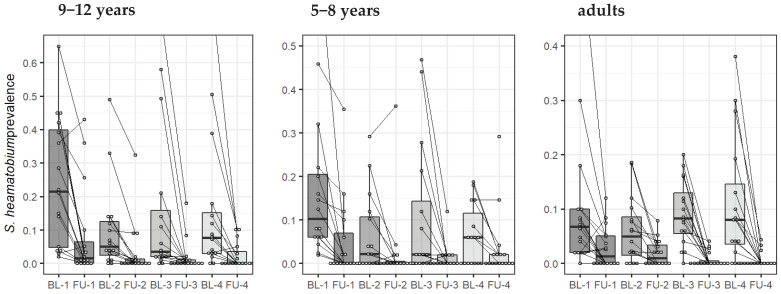
Change in *S. haematobium* prevalence in 64 villages (16 by arm) from baseline (BL) to the first year follow-up (FU), stratified by age group and by study arm (arms 1, 2, 3, and 4). Each line indicates the evolution of one village from baseline to year one, and the boxplots show the distribution by study arm.

Figure 4 shows the change in *S. haematobium* prevalence of the study villages, stratified by population over time. The estimates using inverse probability weighting and for the egg count models are presented in the supplementary Appendix, Figure S1 and Table S1.

### 3.4. Coverage Rate of Preventive Chemotherapy

The treatment coverage by study arm of the initial treatment intervention is summarized in Table 4. The coverage rate by study arm ranged from 65.5% (arm 4) to 82.8% (arm 3). The coverage rate of school-aged children was over 90% in all of the study arms, except for arm 2. Among the 64 localities, 24 villages (37.5%) showed coverage rates above the threshold of 75% recommended by the WHO [16].

## 4. Discussion

The overarching aim of the multiple large-scale SCORE studies was to pursue operational research to deepen our understanding of how to gain and sustain morbidity control of schistosomiasis and achieve the ultimate goal of breaking transmission of the disease [8,9,10,12,13,17]. The current study aimed at characterizing the baseline situation and determining the impact of the first-year intervention with different MDA schedules using praziquantel, combined with chemical snail control in one of the four intervention arms [11]. The study was carried out in the northern and central parts of Côte d’Ivoire, where the transmission of schistosomiasis is seasonal [18]. Our results highlight the *S. haematobium* baseline situation in terms of prevalence and intensity of infection in three different age groups and the effect of the first-year intervention.

Previous studies have shown that *S. haematobium* is the predominant species in the northern and central parts of Côte d’Ivoire [19,20,21]. Our research corroborates these findings and provides a comprehensive update on the distribution of *S. haematobium* over a large part of Côte d’Ivoire. The primary outcome reported here was the change in prevalence among 9- to 12-year-old children one year after the initial intervention between the four study arms.

Data from the initial eligibility and the baseline survey showed that the study was conducted in a low-to-moderate endemic area with prevalence <30% among school-aged children. Our study also revealed that the prevalence was somewhat higher in males than females. This was comparable with studies conducted elsewhere in Côte d’Ivoire and some other parts of the world [22,23,24]. The higher infection level of boys and adults might be explained by socio-cultural and behavioural factors. Indeed, in this part of Côte d’Ivoire, the lack of water in the dry season was compensated for by the creation of small multi-purpose dams for agro-pastoral purposes. Adult males are more often engaged in water-contact activities than females (e.g., fishing, farming, and watering cattle). Boys are engaged more often in recreational activities in the water compared to girls.

Our findings showed a significant decrease in *S. haematobium* infection among school-aged children from a mean prevalence of 16.2% at baseline (of whom 4.0% were heavily infected) to 4.0% at the first year follow-up (of whom 0.7% were heavily infected). Importantly, in the first year follow-up survey, a significant decrease of *S. haematobium* prevalence was observed in all study arms. However, compared to arm 1 (considered as the reference arm with one annual treatment before the peak of the schistosomiasis transmission season), the reduction in prevalence was significantly higher for arm 3 (biannual MDA before and after the peak of the schistosomiasis transmission season) and arm 4 (annual MDA before the peak of the schistosomiasis transmission season, coupled with chemical snail control using niclosamide). These findings were consistent among all age categories. Hence, our results show that after the first year interventions, biannual SBT and CWT, combined with snail control, were able to reduce significantly the overall *S. haematobium* prevalence and infection intensity.

The strong decline in the prevalence and intensity of *S. haematobium* is explained by the administration of praziquantel, which is the drug of choice against schistosomiasis [2,16]. Our results are in line with studies assessing *S. haematobium* prevalence one year post-MDA in Zimbabwe [25], Tanzania [26], Sudan [27], and Senegal [28]. In all of these studies, there was a significant decrease after treatment. However, one year after praziquantel treatment in Nigeria [29], an increase in prevalence was recorded from 2.1% at six months to 7.7% at 12 months post-treatment. Indeed, the higher reduction rate observed in arm 3 can be attributed to the more frequent praziquantel treatment, leading to a shorter time frame between the last MDA and the follow-up survey, and hence, a reduced risk of reinfection. The increase in *S. haematobium* prevalence in two villages despite MDA warrants further investigation, and might be explained by contextual factors, such as low treatment coverage, lack of access to clean water, sanitation and hygiene (WASH), or frequent water contact patterns due to close proximity of these villages to a large dam [13,30,31].

We also found that one year after the intervention, there was a marked decrease in the prevalence and intensity of *S. haematobium* infection in first-grade children and adults. However, there were no significant differences in prevalence and intensity of first-grade children, reflecting a similar effect in this age group of MDA alone or supplemented with chemical snail control. Our results one year after the first round of intervention suggest that the MDA schedules investigated, with or without complementary chemical snail control, in our study had a similar impact on disease transmission at the community level. It will be interesting to monitor the impact over the three years of intervention, determine the differential effect of chemical snail control, and estimate costs of the intervention packages [32]. The longer-term study will provide new evidence for whether any of the interventions are able to interrupt transmission of *S. haematobium* in seasonal transmission foci.

## 5. Conclusions

Our study showed that one year after an initial MDA with praziquantel, there was a significant reduction in the prevalence and intensity of *S. haematobium* in all of the study arms, with a somewhat more pronounced impact when the treatment was given twice before and after the peak of schistosomiasis transmission season. The low rates of reduction observed in some villages and the persistence of some foci despite MDA emphasize that a single round of treatment with praziquantel is insufficient to provide a lasting effect. To reach the ultimate goal of interrupting schistosomiasis transmission, it will be important to monitor changes over longer time frames in different social-ecological settings.

## Figures and Tables

**Table 1:** Baseline characteristics of a cluster randomized trial pertaining to interruption of *S. haematobium* transmission in the northern and central parts of Côte d’Ivoire, stratified by population group.

	School-Aged Children (9−12 years)			
	Arm 1	Arm 2	Arm 3	Arm 4
Villages	16	16	16	16
Total participants	1548	1579	1510	1495
Mean age (years)	10.3	10.2	10.4	10.4
Sex				
Females	628	710	673	671
Males	920	869	837	824
Number of individuals infected with *S. haematobium* (prevalence)	383 (24.8%)	159 (10.1%)	210 (13.9%)	233 (15.8%)
Arithmetic mean infection intensity (eggs per 10 mL of urine)	73.5	55.6	54.1	40.2
Infection intensity				
Light (1−49 eggs per 10 mL of urine)	277 (17.9%)	129 (8.2%)	154 (10.2%)	183 (12.4%)
Heavy (≥50 eggs per 10 mL of urine)	106 (6.8%)	30 (1.9%)	56 (3.7%)	50 (3.4%)
	**First-grade children (5−8 years)**			
Total participants	840	792	806	746
Mean age (years)	6.8	6.9	6.8	6.8
Sex				
Females	408	407	426	391
Males	432	385	380	355
Number of individuals infected with *S. haematobium* (prevalence)	147 (17.9%)	50 (6.3%)	87 (10.8%)	48 (6.6%)
Arithmetic mean infection intensity (eggs per 10 mL of urine)	55.7	64.0	41.7	30.3
Infection intensity				
Light (1−49 eggs per 10 mL of urine)	104 (12.7%)	34 (4.3%)	67 (8.3%)	40 (5.5%)
Heavy (≥50 eggs per 10 mL of urine)	43 (5.2%)	16 (2.0%)	20 (2.5%)	8 (1.1%)
	**Adults (20−55 years)**			
Total participants	734	765	768	761
Mean age (years)	37.9	37.4	37	37.2
Sex				
Females	437	475	463	444
Males	297	290	305	317
Age (20−55 years)	727	761	762	757
Number of individuals infected with *S. haematobium* (prevalence)	85 (11.7%)	46 (6.0%)	70 (10.8%)	83 (11.0%)
Arithmetic mean infection intensity (eggs per 10 mL of urine)	37.2	29.9	11.7	20.3
Infection intensity				
Light (1−49 eggs per 10 mL of urine)	71 (9.7%)	40 (5.2%)	68 (8.2%)	75 (9.9%)
Heavy (≥50 eggs per 10 mL of urine)	14 (1.9%)	6 (0.8%)	2 (0.3%)	8 (1.0%)

**Table 2:** Reduction of prevalence and intensity of *S. haematobium* from baseline to the first year treatment follow-up.

	School-Aged Children (9−12 years)				First-Grade Children (5−8 years)				Adults (20−55 years)			
Variables	Arm 1	Arm 2	Arm 3	Arm 4	Arm 1	Arm 2	Arm 3	Arm 4	Arm 1	Arm 2	Arm 3	Arm 4
**Baseline**												
**Examined**	1544	1573	1506	1469	820	788	804	726	727	761	762	759
**Infected**	383	159	210	233	147	50	87	48	85	46	70	85
**Prevalence (%)**	24.8	10.1	13.9	15.9	17.9	6.4	10.8	6.6	11.7	6.0	9.2	11.0
**Village-level AM infection intensity (eggs per 10 mL of urine)**	17.9	5.7	8.4	6.2	9.6	4.1	4.5	2.0	4.0	2.0	1.1	2.1
**Individual-level AM infection intensity (eggs per 10 mL of urine)**	73.5	55.9	54.1	40.2	55.7	64.0	41.7	30.4	37.2	29.9	11.7	20.0
**Year 1**												
**Examined**	1538	1561	1510	1487	789	805	774	777	596	694	626	590
**Infected**	124	55	29	33	41	21	10	27	18	13	5	4
**Prevalence (%)**	8.1	3.5	1.9	2.2	5.2	2.6	1.3	3.5	3.0	1.9	0.8	0.7
**Village-level AM infection intensity (eggs per 10 mL of urine)**	3.7	2.3	0.2	0.6	1.1	2.1	0.2	2.9	0.8	0.8	0.1	0.2
**Individual-level AM infection intensity (eggs per 10 mL of urine)**	46.1	65.3	9.4	26.4	21.8	76.1	18.1	84.3	23.9	50.0	19.2	23.3
**Absolute difference prevalence (%-points)**	−16.7	−6.6	−12.0	13.7	−12.7	−3.7	−9.5	−9.5	−8.7	−4,1	−8.4	−8.4
**Relative difference prevalence (% change)**	−67.3	−65.3	−86.3	−86.1	−70.9	−58.7	−88.0	−88.0	−74.3	−68.3	−91.3	−91.3
**Egg reduction rate (%)**	−79.3	−59.6	97.6	90.3	−88.5	−48.8	95.5	−45.0	−80.0	60.0	90.9	93.6

**Table 3:** GEE logistic model to assess OR and aOR between arms at the one-year follow-up for all age groups. aOR, adjusted odds ratio; CI, confidence interval; OR, odds ratio. The adjusted model includes age, sex, and cluster level baseline prevalence as additional covariates and is weighted to account for different numbers of observations in each cluster.

Age Group	Comparison	Difference in Prevalence	
		OR (95% CI)	aOR (95% CI)
**9** **−** **12 years**	Arm 2 vs. arm 1	0.42 (0.10-1.80)	1.03 (0.34−3.07)
	Arm 3 vs. arm 1	**0.22 (0.05−0.95)**	**0.19 (0.08−0.48)**
	Arm 4 vs. arm 1	**0.26 (0.08−0.85)**	**0.23 (0.06−0.87)**
**5** **−** **8 years**	Arm 2 vs. arm 1	0.49 (0.08−3.01)	1.38 (0.17−11.10)
	Arm 3 vs. arm 1	0.24 (0.06−1.03)	0.39 (0.10−1.54)
	Arm 4 vs. arm 1	0.33 (0.08−1.31)	0.91 (0.20−4.23)
**20−55 years**	Arm 2 vs. arm 1	0.61 (0.25−1.52)	0.88 (0.36−2.12)
	Arm 3 vs. arm 1	**0.26 (0.08−0.81)**	**0.32 (0.11−0.96)**
	Arm 4 vs. arm 1	**0.22 (0.06−0.79)**	**0.21 (0.07−0.64)**

**Table 4:** Praziquantel coverage rate by treatment round by study arm for the first year intervention.

Treatment	Annual MDA Before the Peak Transmission Season			Annual MDA After the Peak Transmission Season	
Year 1	Arm 1	Arm 3	Arm 4	Arm 2	Arm 3
MDA period	December 2015	December 2015	December 2015	April 2016	April 2016
No. of villages treated	16	16	16	16	
School-aged children treated	4988	7086	5018	4206	7024
Total number of school-aged children	5145	7440	5381	5572	7440
Treatment coverage in school-aged children (%)	96.9	95.2	93.3	75.5	94.4
Adults treated	8942	12,671	12,700	9897	12,992
Total number of adults	12,081	20,267	21,650	15,809	20,978
Treatment coverage in adults (%)	74.0	62.5	58.6	62.6	62.0
Total no. of participants treated	13,930	19,757	17,718	16,391	23,849
Total participants	17,226	27,707	27,031	21,381	28,418
Overall coverage (%)	80.9	71.3	65.5	76.7	83.9

## Supplementary Materials

The following are available online at https://www.mdpi.com/2414-6366/6/1/7/s1, Figure S1: Logistic GEE models with inverse probability weighting, Table S1: GEE negative binomial model.
