# Supplementary material for: Baseline and Impact of First-Year Intervention on Schistosoma haematobium Infection in Seasonal Transmission Foci in the Northern and Central Parts of Côte d’Ivoire
Source: Trop Med Infect Dis. 2021 Jan 8;6(1):7. doi: 10.3390/tropicalmed6010007 (PMC7838999; doi:10.3390/tropicalmed6010007)
Supplement: Supplementary file 1 [file tropicalmed-06-00007-s001.pdf]

Supplementary Materials

To account for imbalance in baseline prevalence, clusters were weighted in the analysis using inverse probability weights (estimated with R's ipw package). Otherwise, the models are similar to the unadjusted logistic GEE models presented in table 3. Black symbols, (dashed) lines and numbers represent the IP-weighted odds ratios, confidence intervals and prevalences. Gray symbols and lines present the unadjusted models forcomparison.

Interpretation example: panel top left (9 to 12 years). The observed prevalences at baseline in arm1 was 24.8% and 10.1% in arm 2. After applying IP-weights, the weighted prevalence in arm 1 was 18.1% at baseline and 18.4% in arm 2. After one year the observed prevalences were 8.1% in arm 1 compared to 3.5% in arm 2 and the weighted prevalences 4.4 and 8,9, respectively. Similarly, the unadjusted odds ratio is below 1 (about 0.4). Contrary, the weighted odds ratio is above 1 (about 1.7). In both analyses the 95% confidence interval includes unity, indicating that the estimated difference isnot statistically significant.

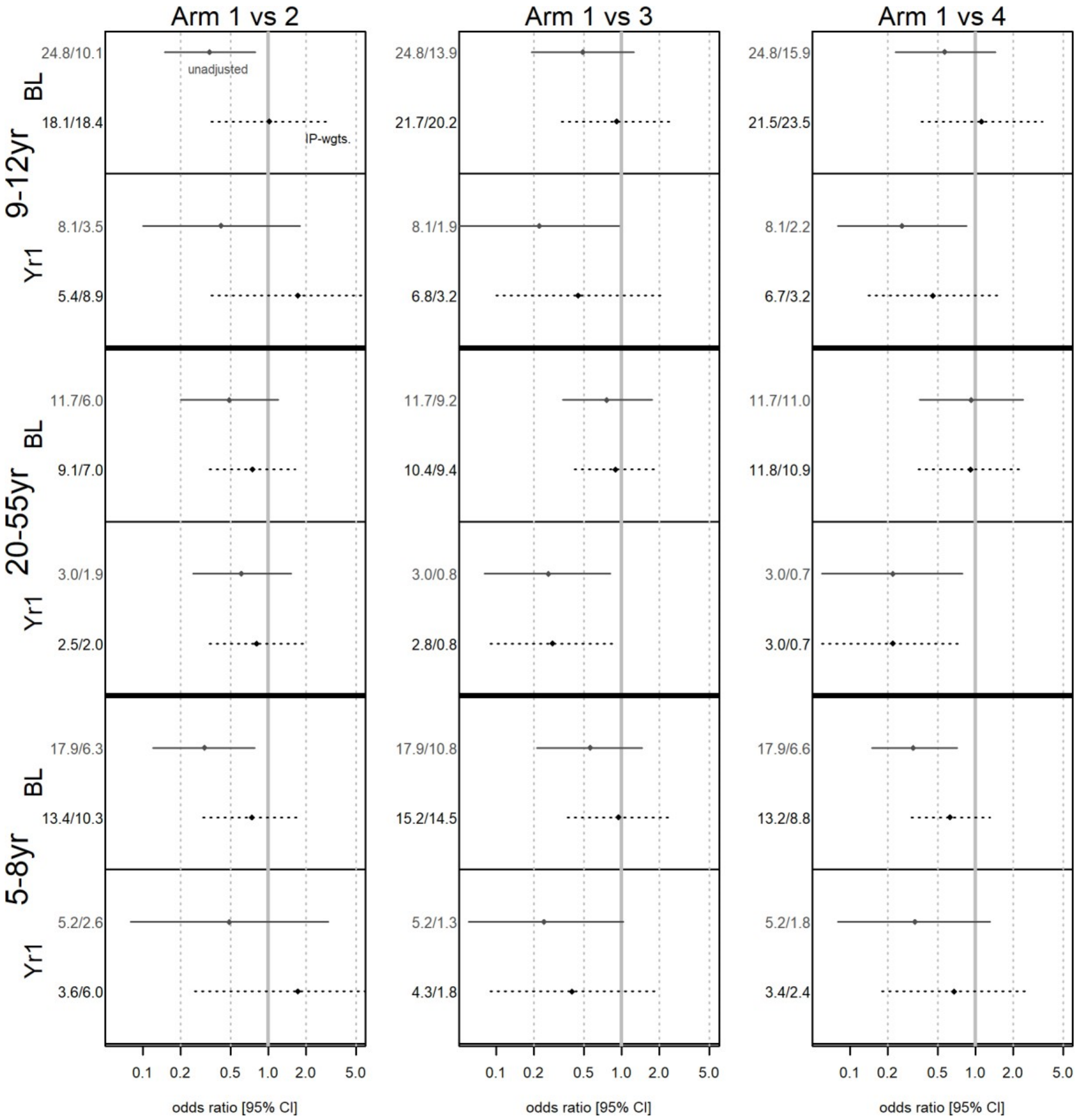

Figure S1. Logistic GEE models with inverse probability weighting.

Table S1. GEE negative binomial model.

Age Group	Comparison	Difference in Egg Counts	
		Unadjusted CR (95% CI)	Adjusted CR (95 % CI)
**9-to-12-year**	Arm 2 vs Arm 1	0.62 (0.09-4.33)	0.63 (0.14-2.77)
	Arm 3 vs Arm 1	**0.05 (0.01-0.24)**	**0.22 (0.06-0.86)**
	Arm 4 vs Arm 1	**0.16 (0.04-0.72)**	0.51 (0.10-2.47)
**5-to-8-year**	Arm 2 vs Arm 1	1.75 (0.22-14.05)	0.72 (0.10-5.18)
	Arm 3 vs Arm 1	**0.21 (0.04-1.00)**	0.66 (0.12-3.78)
	Arm 4 vs Arm 1	2.58 (0.53-12.51)	5.36 (0.74-38.97)
**20-to-55-year**	Arm 2 vs Arm 1	0.29 (0.06-1.58)	0.40 (0.12-1.35)
	Arm 3 vs Arm 1	**0.07 (0.02-0.32)**	0.36 (0.07-1.89)
	Arm 4 vs Arm 1	0.35 (0.07-1.67)	**0.03 (0.01-0.16)**

Estimation of differences in infection intensity of *S. haematobium* (eggs per 10 mL) between arms at year 1 for all the age groups. CR: count ratio, 95% CI = 95% confidence interval. The adjusted model includes age, sex and cluster level baseline prevalence as additional covariates and is weighted to account for different number of observations in each cluster.
